# Assessment of suturing and scaling skills of periodontology and oral medicine residents by OSATS method: a pilot study

**DOI:** 10.1186/s12909-023-04875-0

**Published:** 2023-11-21

**Authors:** Fahimeh Rashidi Maybodi, Fatemeh Keshmiri, Maryam Kazemipoor, Fatemeh Owlia

**Affiliations:** 1 Periodontics Department, Dental faculty, Shahid Sadoughi University of Medical Sciences, Yazd, Iran; 2 Faculty of Public Health, Shahid Sadoughi University of Medical Sciences, Yazd, Iran; 3 Department of Endodontics, School of Dentistry, Shahid Sadoughi University of Medical Sciences, Yazd, Iran; 4 Department of Oral and Maxillofacial Medicine, School of Dentistry, Shahid Sadoughi University of Medical Sciences, Yazd, Iran

**Keywords:** Periodontics, Checklist, Suturing, OSATS, Oral medicine, Scaling

## Abstract

**Introduction:**

Updating the method for evaluating suturing and scaling skills in dental education has attracted relatively little attention and there is no consensus to what should be assessed and how. The purpose of this study was to investigate the applicability of the Objective Structured Assessment of Technical Skill (OSATS) method for these two basic skills, the possible association between the scores and demographic factors, and the level of satisfaction of residents with this method.

**Methods:**

All six periodontics and three oral medicine residents were recruited by census method and video-recorded while performing a simple interrupted suture, a figure eight suture and scaling on a model. Then, the videos were evaluated independently via a checklist and a global rating scale (GRS) by two expert raters. Agreement between raters and residents’ satisfaction were evaluated. Correlation between demographic factors of participants and scores was also assessed. T-test and linear regression analysis were used.

**Results:**

There was no significant difference between the scores based on the views of the two raters for each of the checklist (ICC = 0.99, CI = 0.96–0.99, *P* < 0.001) and GRS (ICC = 0.97, CI = 0.86–0.99, *P* < 0.001). Linear regression showed no correlation between gender and scores but periodontics major and higher year of education showed correlation with higher scores.

**Conclusion:**

Considering the excellent agreement between raters in using both the checklist and GRS components of OSATS, and satisfaction of 88% the residents with this method, it seems to be able to provide a reliable assessment.

**Supplementary Information:**

The online version contains supplementary material available at 10.1186/s12909-023-04875-0.

## Introduction

Assessment is an important component of education because it is used to identify students’ abilities in order to achieve educational goals [1]. Assessing the clinical competence of students is one of the most main tasks for faculty members and educators in medical sciences programs [2]. Clinical evaluation is more difficult because different variables that are out of control can affect it [3]. Traditional written and verbal exams only measure clinical knowledge while objective methods evaluate both knowledge and skill [4, 5]. In addition, existing clinical knowledge is not free from validity and reliability limitations. Structured methods can modify some of these limitations [5]. Apart from the reliable assessment of skills, the psychometric characteristics of an evaluation method and getting feedback from trainees should also be considered when choosing a method [6]. In objective structured methods, Individuals’ skills in performing tasks are objectively assessed as an effort to minimize the bias of examiner subjective judgment [7]. Common structured evaluative tools include the OSCE (Objective Structured Clinical Examination) which is a general title for clinical examinations to evaluate the skills objectively via organized stations [8]. One type of OSCE is the Objective structured assessment of technical skills (OSATS), first used in 1990 by the department of Surgical Education Research at the University of Toronto for surgical residents and pursued two goals: (1) Evaluating practical skills on the model and outside the operating room (2) Developing the validity and reliability of practical skills assessment tools [9]. In OSATS, residents must pass several stations over a limit period of time. This test consists of two components: a checklist for evaluating the steps of the technique or procedure (Operation-Specific Check list) and a detailed global rating scale (GRS) [7].

Most of the studies which used this method, were in specialized medical fields [10, 11] and for practical skills which traditionally were assessed subjectively by the supervisor in the operating room [9]. In the field of dentistry, especially in the specialized field, studies are very limited. Caminiti et al. study in 2021, conducted as a pilot study on maxillofacial surgery residents [6], can be mentioned as an example. Undoubtedly, there is no absolutely perfect method in assessing the basic clinical skills of periodontics or oral medicine residents such as suturing and scaling, because each tool has its own advantages and limitations that make it applicable on some situations. The characteristics of structured evaluation methods are to be as similar as possible to the real situation, to be more objective than other tools, to use the same questions for all students, and to have high reliability and validity.

In the samples we investigated, the current assessment was based on a logbook that was not accurate in recording practical skills due to limitations such as the unavailability of feedback discussion opportunities or assess learners’ competence [12]. The purpose of this pilot study was to explain our experiences regarding the design and implementation of the OSATS, as an innovative effort in the direction of representing a promising novel evaluation of two primary practical skills (1. Scaling 2. suturing) in the field of complementary dental education in periodontics and oral medicine departments of Shahid Sadoughi University of Medical Sciences, Yazd, Iran, from April 2021 to August 2021. A secondary objective of the authors was to sensitize dental examiners, professors and residents to OSATS. So, the following main research questions were considered: (1) Was there inter-rater agreement in the use of GRS and Checklist? (2) Was there any difference in the obtained scores on the basis of demographic factors of the residents? Were the residents satisfied with the way it was held and also the outcome?

It should be noted that no similar study has been done before in Yazd dental school or in any other dental schools in Iran. Dentistry students are traditionally become familiar with suturing and scaling skills in a simulated environment using animal models or synthetic materials [13]. Therefore, the same tools were used in the design of this method.

## Materials and methods

### Ethical approve

This study was approved by the ethics committee of Shahid Sadoughi University of Medical Sciences (IR.SSU.REC.1398.032) and written informed consent was obtained from all participants. All methods were performed in accordance with the 1964 Helsinki.

### Designing phase

Exam planning steps was conducted in six steps by a panel of experts consisted of 2 periodontists and 2 oral medicine specialists:

- Development of blueprint of examination and Agreement on the contents of the exam.
- Designing stations and determination of the score of each station.
- Designing and validation confirmation of checklists and questionnaire to assess residents’ skills and perspectives.
- Education of Raters.
- Exam descriptions and instruction to residents and staff involved in the exam.
- Reviewing stations and exam process.

### Subjects

In Iran, the periodontics and oral medicine residency program lasts three years. All Six residents studying in the first to third years of the periodontics and all 3 oral medicine residents (totally 9 participants) were enrolled by census method (all students studying in 2021–2022 interval, were recruited) in this quasi-experimental study.

### Implementation phase

Before the exam, a briefing session was held for the professors (two periodontists) present at the exam and a separate session for the residents on how to conduct the exam.

**Fig. 1:**
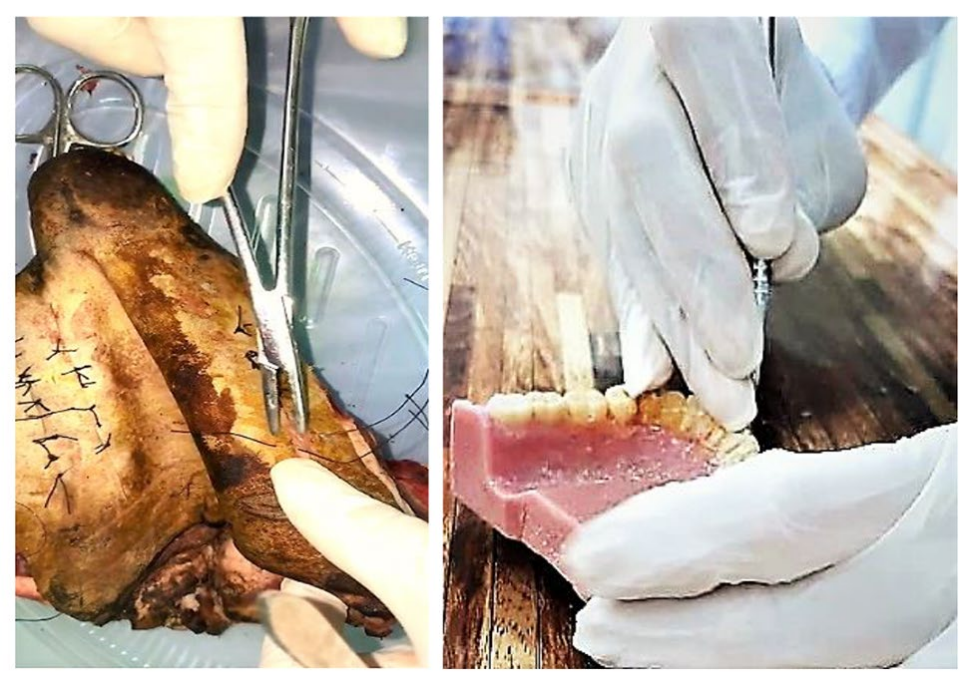
(**a**) Suturing, (**b**) Scaling

In this exam, a total of two stations were designed: (1) suturing (figure of eight and simple sutures on the animal model: sheep scalp) (Fig. 1a), (2) Scaling (on artificial model) (Fig. 1-b).

The purpose of implementing these two stations was to evaluate two common and necessary basic technical skills in both specialized fields (periodontics and oral medicine). One skill was selected from the category of skills needed during invasive interventions such as pocket elimination surgery or preparation of excisional biopsies, etc. and one skill was selected from the category of non-surgical technical skills (scaling with the aim of restoring periodontal health in systemically healthy patients or patients with systemic conditions need to observe pre- and post-treatment considerations).

The instructions for each station and what was exactly asked from the residents, were installed as a guide on the table next to the instruments needed by that station.

Residents were asked to perform a simple loop suture and a figure of eight suture on the sheep tissue over a defined time (1 min for each) with the help of a silk suture number 4 − 0 and a 19 mm needle by using same type of needle-holder and scissors.

At the second station, dark yellow adhesive wax was already poured as a substitute for calculus in a way that be visible to the raters and was shaped at the height of 3–4 mm in the gingival margin area of 6 anterior mandibular teeth and the residents were asked to completely remove pseudo- supra gingival calculus with Universal curette scalers. The time allocated to this station was 5 min.

### Evaluation phase

Two scoring methods were designated for each station:

1. Two separate task-specific checklists, an 8-item one for suturing station and a 7-item one for Scaling station. (Table 1A in Appendix)
2. Two global rating scales (GRS) for each skill (Tables 2A and 3A in Appendix)

Since suturing skill had already been assessed with the OSATS method in the medical field, fortunately a standard checklist and a pre-prepared GRS were available for this skill [10], but for scaling, these two components were newly designed and implemented [14].

In explaining how to evaluate the exam, it should be noted that in addition to the supervision of 2 clinical professors in the periodontics department on the correct execution of designed and approved stations, videos from residents were prepared during the exam by a third party in a blind manner that only models and two hands of each resident could be seen to eliminate the influence of having a previous mindset unwanted effect on the scoring. One periodontist and one oral medicine specialist (other than 2 examiners) viewed each video in its entirety first. Then each rater viewed the same video again while scoring the checklist. Then the GRS was scored at the end of the third round of video review.

In the checklist scoring, each type of suturing had a total 40 points and the scaling skill had 35 points, that is, for each item from 1(not done) to a maximum of 5 points (done completely). The point ‘3’ was considered for ‘done but not completely’ choice. In the global rating scale, domains were graded on a Likert scale from 1 to 5, there were explicit explanations in points 1, 3, and 5. In global rating method, each rater gave one mean score for two types of suturing (total suturing score) and a score was also reported for scaling. The evaluators also gave verbal feedback to the participants about their weak points after the exam.

Residents’ satisfaction was also assessed with the help of a questionnaire on a Likert scale with five options: strongly agree, agree, have no opinion, disagree and strongly disagree for each item. Two weeks later, we conducted interviews and informally asked residents if identifying their weaknesses during the exam helped them improve their suturing and scaling skills.

### Statistical analysis

Normality distribution of data was checked by Shapiro-Wilk test. Data was summarized by descriptive test (Mean, SD). T-test was used for comparing scores in groups and Linear regression analysis was done separately for assessing the correlation between scores and demographic factors. Interclass coefficient (ICC) was used for evaluating inter-rater agreement. *P* < 0.05 was considered statistically significant. Correlation between residents’ overall satisfaction and their scores was also assessed by calculating Spearman’s coefficient.

## Results

Seven post-graduate students were female and two were males. Six out of nine were post-graduate students of periodontics and three were oral medicine post-graduate students.

All 9 residents participated in the exam voluntarily and completed the satisfaction questionnaire at the end of the exam.

According to T-test, no significant difference was seen between given mean scores based on two raters. The mean scores of each rater for each skill according to two different assessment tools (Global rating and checklist), were given in Table 1.

**Table 1:** Comparison of mean scores based on two different raters

Assessment Tool	Skills	Rater 1 mean score ± SD	Rater 2 mean score ± SD	*p*-value
GRS	Simple suture	31.5 ± 0.8	32.3 ± 2.8	0.54
	Figure 8 suture	29.9 ± 0.9	30.2 ± 0.6	0.76
	Scaling	25.5 ± 3.1	26.4 ± 3.1	0.55
CHECKLIST	Simple suture	32.9 ± 1.5	33.3 ± 1.5	0.83
	Figure 8 suture	30.2 ± 1.86	29.7 ± 1.84	0.83
	Scaling	30.8 ± 0.8	30.8 ± 0.9	1.00

T-test

The agreement between two raters was also assessed by estimation of an overall inter-rater intra-class correlation coefficient for checklist (ICC = 0.99, CI = 0.96–0.99, *P* < 0.001) and GRS (ICC = 0.97, CI = 0.86–0.99, *P* < 0.001) components, which both can be interpreted as excellent.

According to T-test, Table 2 shows that there was no difference between scores of females and males in none of the skills. Comparison of residents’ scores in the basis of Major is showed in Table 3 and significant differences were starred. The only superiority for periodontics residents agreed by both evaluators, was Fig. 8 suturing on the base of the checklist.

**Table 2:** Comparison of residents’ scores in the basis of gender

Assessment Tool	Skills	Rater’s code	Gender	Mean score ± SD	*p*-value
	Simple suture	1	F	31.3 ± 2.4	0.58
			M	32.5 ± 3.5	
		2	F	31.1 ± 5.3	0.728
			M	27.0 ± 7.1	
GRS	Figure 8 suture	1	F	29.7 ± 2.8	0.75
			M	30.5 ± 3.5	
		2	F	30.3 ± 2.0	0.85
			M	30.0 ± 0	
	Scaling	1	F	25.1 ± 3.4	0.86
			M	27.0 ± 1.4	
		2	F	26.3 ± 3.1	0.89
			M	27.0 ± 4.2	
	Simple suture	1	F	32.8 ± 4.9	0.97
			M	33.0 ± 4.2	
		2	F	33.4 ± 5.1	0.91
			M	33.0 ± 1.4	
CHECKLIST	Figure 8 suture	1	F	31.1 ± 5.4	0.39
			M	27.0 ± 7.1	
		2	F	30.1 ± 5.2	0.66
			M	28.0 ± 8.5	
	Scaling	1	F	30.9 ± 2.3	0.86
			M	30.5 ± 3.5	
		2	F	30.9 ± 3.1	0.89
			M	30.5 ± 3.5	

T-test

**Table 3:** Comparison of residents’ scores in the basis of Major

Assessment Tool	Skills	Rater’s code	Major	Mean score ± SD	*p*-value
	Simple suture	1	Perio	32.7 ± 2.2	0.051
			Med	29.3 ± 1.1	
		2	Perio	33.7 ± 1.0	0.029*
			Med	29.7 ± 3.5	
GRS	Figure 8 suture	1	Perio	31.1 ± 2.5	0.042*
			Med	27.3 ± 1.1	
		2	Perio	30.6 ± 1.9	0.323
			Med	29.3 ± 1.1	
	Scaling	1	Perio	26.7 ± 3.0	0.583
			Med	23.3 ± 2.3	
		2	Perio	28.0 ± 2.5	2.08
			Med	23.3 + 1.1	
	Simple suture	1	Perio	35.3 ± 3.0	0.007*
			Med	28.0 ± 2.0	
		2	Perio	35.0 ± 4.5	0.118
			Med	30.0 ± 2.0	
CHECKLIST	Figure 8 suture	1	Perio	33.7 ± 2.6	0.000*
			Med	23.3 ± 1.1	
		2	Perio	33.2 ± 2.0	0.000*
			Med	22.7 ± 1.1	
	Scaling	1	Perio	31.8 ± 2.1	0.051
			Med	28.7 ± 1.1	
		2	Perio	32.5 ± 1.6	0.002*
			Med	27.3 ± 1.1	

T-test

The results of linear regression analysis for the correlation between the scores of each component (checklist (CH) & Global Rating Scale GRS) and demographic factors were shown in Table 4 and significant correlations were starred. There was no correlation between gender and scores.

**Table 4:** Correlation of scores with demographic factors

Variables	Major			Year of education			Gender		
	*B*	*t*	*p*-value	*B*	*t*	*p*-value	*B*	*t*	*p*-value
CH.1 total scores	25.23	17.93	0.001*	6.96	8.24	0.001*	-1.43	-0.96	0.37
CH.2 total scores	25.65	12.8	0.001*	7.50	6.28	0.002*	-0.11	-0.054	0.95
GRS.1 total scores	15.37	7.58	0.001*	6.07	4.99	0.004*	4.98	2.32	0.06
GRS.2 total scores	14.3	10.11	0.001*	5.91	6.96	0.001*	2.24	1.49	0.19

Multivariate linear regression

The results showed that 78% of the residents were completely satisfied with how the exam was performed, 11% were satisfied and 11% had no opinion. 89% of the residents completely agreed and 11% of them agreed that this exam provided an opportunity for them to learn more. 78% strongly believed that the exam showed them their weaknesses, and 22% agreed with the above. 22% of residents thought that using this method is stressful. More details are given in Table 5. There was no correlation between overall satisfaction of residents (item No.11) from performance of this exam and their scores (Table 6).

**Table 5:** Residents’ satisfaction with how the exam was held

Questions	Strongly agree	Agree	No opinion	Disagree	Strongly disagree
1. The duration of each station was appropriate	4(45%)	5(55%)			
2. The exam was well administered	5(55%)	4(45%)			
3. The instructions for each station were clear	6(67%)	3(33%)			
4. The equipment provided was in line with the requirements of each station	5(55%)	4(45%)			
5. The requirements in all stations were in accordance with the training given in the practical courses	6(67%)	3(33%)			
6. Assessment with OSATS method promotes the practical knowledge of residents	5(55%)	4(45%)			
7. This exam was an opportunity to learn	8(89%)	1(11%)			
8. This method identified the weak points of the residents in performing the skills	7(78%)	2(22%)			
9. Assessment with OSATS method increases the stress caused by the exam in the residents	----	2(22%)	4(45%)	2(22%)	1(11%)
10. This method henceforth be used as part of the evaluation of practical courses in the residency	3(33%)	5(56%)	1(11%)		
11. Overall the performance of the exam was satisfactory.	7(78%)	1(11%)	1(11%)		

**Table 6:** Correlation of scores with residents’ overall satisfaction

Variables	Overall satisfaction	
	Coefficient	*p*-value
CH.1 total scores	0.47	0.192
CH.2 total scores	0.42	0.255
GRS.1 total scores	0.65	0.056
GRS.2 total scores	0.59	0.090

Spearman’s

### Post-hoc analysis for sample size

In the post-hoc analysis, considering the significance level of 0.05 and the test power of 95%, and according to the agreement coefficient obtained in this study, which was more than 0.95, the total sample size was estimated to be 7.

## Discussion

Choosing an appropriate assessment method leads to higher quality learning outcomes [15]. In line with this general goal, in the present study, the following main research questions about OSATS method were answered: whether any demographic factor affected the scores or not, secondly how was the inter-rater agreement between the evaluators and finally were the residents satisfied with this method or not.

As mentioned earlier, there was a limitation of similar studies in the field of dental skills. Therefore, it was inevitable to mention studies in the field of medicine or nursing. Another issue is that, in the studies of the medical field, the evaluation of suturing skill had been done and the possibility of comparison, although limited, was available but in the case of scaling, it was the first time that the OSATS method was designed and implemented in a study, and therefore, unfortunately, there was no room for comparative discussion about this skill.

The agreement between the two raters for both the checklist and the GRS can be interpreted as excellent. In some previous studies, no further evaluation of interrater reliability was addressed [9, 10]. Chang et al. [11] also reported inter-rater agreement for OSATS as high, which was consistent with our study.

However, it was expected that residents in the higher years could obtain higher scores, but according to T-test analysis, there was no significant difference between the scores of post-graduates of different years in all evaluated items (simple suture, figure of eight suture and scaling). On the other hand, the results of linear regression indicated higher scores in residents of higher years, which met the initial expectation. In Niitsu et al.‘s study, which examined the general skill of residents in performing simple surgeries such as appendectomy to difficult surgery such as hepatectomy, the average score of the global rating scale improved with year of experience [9]. In the study of Al-Qahtani et al., the general trend of improving grades in the higher year of education was also shown among otolaryngology residents performing tracheostomy using OSATS [16]. It should be noted that in these studies, skills of a different nature and higher level of difficulty were examine, compared to the present study. In Chang et al.‘s study, junior residents had a lower score in suturing during laparoscopy than senior residents but this was not statistically significant [11]. No correlation was also found between dermatology residents’ suturing scores and the number of surgical rotation months in Alam et al. study [10].

In a comparison with the help of t-test, periodontics residents gained higher scores than oral medicine residents in figure of eight suturing using checklist according to both raters but in other skills or according to GRS, such an agreement was not seen in their superiority. This may indicate that oral medicine residents seems to have the necessary competence to perform a fully scaling for their patients and suturing during limited surgeries, such as preparing an excisional biopsy or extracting several adjacent teeth. In contrast, linear regression showed the superiority of periodontics residents in overall scores of these two very basic skills. In this study, two majors involved in surgical activities were selected, but in other previous studies, participants were selected from the same field. Therefore, it was not possible to compare this variable (specialty field) with the literature.

There is a preconceived notion that male students are significantly more confident than females in general aspects of practical skills [17] which may effect on the quality of their practice, although some studies also reject such an assumption [18]. In the present study, according to T-test analysis, there was no significant difference in the scores of male and female residents neither in the field of suturing nor in the field of scaling. Such an issue was not considered in similar studies. Linear regression analysis also showed no correlation between gender and scores.

The initial spark of the design of this study was the feedback that we received during the past semesters about the residents’ dissatisfaction with the traditional method, and this issue was the main motivation for all of them to participate in this study. All the residents who were being trained, were included in the study by census method, and therefore the possibility that only residents who were fully confident in their skills were included in the study is not raised. However, eight out of 9 residents (88%) stated that the overall performance of the exam was satisfactory. In the present study, only 22% of residents thought that using this method is stressful. There was no correlation between overall satisfaction and residents’ scores which may indicate the internal desire of the residents to use the new method by the professors to evaluate them apart from the grades of this exam. Pishkar Mofrad et al [2]. showed that the majority of nursing students considered OSATS assessment to be a stressful method which was not in line with the above research. Probably the reason for this discrepancy is the difference nature of field of study, type of assessed skills, the design and the number of stations. Regarding the use of objective structured evaluations in the field of dentistry, in the study of BasirShabestari et al. [1], about 73% of the undergraduate dentistry students considered the OSCE method to be stressful which is not consistent with our study. This is probably due to the greater readiness of residents compared to general dentistry students to take structured examinations.

About 89% of nursing students in Mansoorian study [7] thought that OSATS can evaluate the student’s weaknesses better than traditional methods that is consistent with our results which showed that 100% of residents considered this method as a good identifier of their weak points in performing the skills. In Chisthi et al. study, majority of the medical students felt that the assessment was more objective when OSATS tool was used for suturing skill [19].

In order to provide assessment tools for competency-based training, we must continue to develop improved valid reliable tools with a high degree of discrimination and as little cost as possible for surgical skills. The acceptable reliability of this method has been confirmed in most studies [19–21]. Vaidya et al. in 2020 in a systematic review on 303 studies which had used any assessment tool in any surgical specialty in medical field, among many valid tools available for assessing practical skills, introduced OSATS as the most common technique which has been validated by using Messick’s validity framework [22]. According to a systematic review by Hatala et al. in 2015, OSATS can be considered as a valid and reliable method for scoring and extrapolation of the continuous educational process during a course if accompanied by giving feedback to learners but if it is intended to be used in decisions with higher risk such as certification at the end of residency course, there is not enough evidence in the studies to generalize the results [23].

In choosing an appropriate method, inter-rater reliability may also be considered as part of its generalizability [23]. In this study, no significant difference was observed between two raters based on the checklist and the GRS in all three items, which may be a promise for its applicability in dental departments. In confirmation of this, the interclass coefficient (ICC) between raters also showed their excellent agreement (more than 95%) based on the overall scores in both Checklist & GRS. The agreement between two raters, one of whom is a periodontist and the other an oral medicine specialist, can indicate the correct selection of items included in the checklists and GRS_s_, by the panel of experts in designing phase. Considering the above-mentioned contents and the results of the present study, OSATS can be suggested as a reliable, quick and cheap method in routine dentistry assessments.

This method consists of two components and these two assessment tools follow somewhat different and complementary goals. In the checklist, the overall performance of the procedural steps and compliance with the correct order are mainly emphasized. On the other hand, the global rating scale emphasizes the quality of technique execution. To achieve a good score on the OSATS, a skill must be performed not only correctly, but skillfully, for example without unnecessary pauses or extra movements [24]. Therefore, our suggestion is to use both even in cases of lack of time or other implemental limitations.

### Limitations

The small sample size was the main limitation of the present study, although in the post-hoc analysis, the selected sample size was finally evaluated as sufficient. Another limitation of this study was the lack of prior familiarity of residents with the OSATS method, which was conducted for the first time in the faculty, which may have unintentionally affected the residents’ performance, even though they had been previously explained how to take the test.

### Further direction

This study examined the steps and preparations necessary to implement OSATS and highlights the positives of this method. Studies with larger sample size and in a multi-center manner will be useful for the generalizability of this method. Also, conducting a comparative evaluation of periodontics residents with undergraduates or with periodontal specialists in future studies may bring interesting results.

## Conclusion

Considering the inter-rater reliability and the overall satisfaction of the residents with this method, OSATS seems to be able to provide a reliable assessment and is recommended for wider use in the dental education.

### Electronic supplementary material

Below is the link to the electronic supplementary material.

Supplementary Material 1

## Data Availability

Derived data supporting the findings of this study are available from the corresponding author on request.
