# Supplementary material for: Assessment of suturing and scaling skills of periodontology and oral medicine residents by OSATS method: a pilot study
Source: BMC Med Educ. 2023 Nov 21;23:889. doi: 10.1186/s12909-023-04875-0 (PMC10664488; doi:10.1186/s12909-023-04875-0)
Supplement: Supplementary file 1 — Supplementary Material 1 [file 12909_2023_4875_MOESM1_ESM.docx]

**Appendix:**

**Table 1A: Checklists for suturing and Scaling**

| **Items** | **Not done**  (1 point) | **Done but not completely**  (3 points) | **Done completely**  (5 points) |  |
| --- | --- | --- | --- | --- |
|  | 1. Correct placement of needle in holder |  |  |  |
| **Suturing** | 1. Correct angle insertion of needle into the tissue (90 degree) |  |  |  |
|  | 1. Equal bites on either sides of wound |  |  |  |
|  | 1. Correct entry and exit points of needle |  |  |  |
|  | 1. Smooth passage of needle, no hesitancy |  |  |  |
|  | 1. Careful handling of tissue with forceps |  |  |  |
|  | 1. Correct knotting |  |  |  |
|  | 1. Correct distance between sutures |  |  |  |

|  | 1. Stable grip on the instrument |  |  |  |
| --- | --- | --- | --- | --- |
| **Scaling** | 1. Proper rest while applying force |  |  |  |
|  | 1. Proper adaptation of the instrument on the tooth surface |  |  |  |
|  | 1. Correct alignment of the instrument |  |  |  |
|  | 1. Correct motion of the instrument |  |  |  |
|  | 1. Complete removal of calculus (wax) |  |  |  |
|  | 1. Checking the smoothness of all surfaces * |  |  |  |

- * Using pigtail explorer

**Table 2A: Global Rating for suturing**

| **Item** | **1** | **2** | **3** | **4** | **5** |
| --- | --- | --- | --- | --- | --- |
| **Respect for tissue** | Frequently used  unnecessary  force on tissues or  caused damage by  inappropriate instrument use |  | Careful handling  of tissue but occasional inadvertent damage |  | Consistently handled tissues appropriately  with minimal damage |
| **Time and motion** | Many unnecessary moves |  | Efficient time and motion but some unnecessary moves |  | Clear economy  of movement and maximum efficiency |
| **Instrument handling** | Repeatedly makes tentative or awkward moves with instruments |  | Competent use of instruments, but occasionally awkward |  | Fluid movements |
| **Suture training** | Awkward and unsure with poor knot tying, and inability to maintain tension |  | Competent suturing with good knot placement and appropriate tension |  | Excellent suture control with correct suture placement and tension |
| **Knowledge of**  **procedure** | Inefficient knowledge of procedure. Looked unsure and hesitant |  | Knew all important steps of procedure |  | Demonstrated familiarity of all steps of procedure |
| **Final product** | Final product of unacceptable quality |  | Final product of average quality |  | Final product of superior quality |
| **Overall performance** | Very Poor |  | Competent |  | Very Good |

| Item | 1 | 2 | 3 | 4 | 5 |
| --- | --- | --- | --- | --- | --- |
| Correct adaptation of instrument | Incorrect adaptation with tooth surface |  | Lose adaptation occasionally |  | Continuous adaptation of the blade with the tooth surface |
| Proper angulation of the instrument | Unwanted calculus burnishing because of sharper or wider angulation |  | Incorrect change of angulation occasionally |  | Correct angulation (45-90 degree for scaling) |
| Time and motion | Many unnecessary moves |  | Efficient time and motion but some unnecessary moves |  | Clear economy  of movement and maximum efficiency |
| Instrument handling | Long and light strokes |  | Competent use of instruments, but occasionally awkward |  | Short and strong strokes |
| Final outcome | many detectable calculus remained |  | Little amount of calculus remained |  | All detectable supra calculus removed |
| Overall performance | Very Poor |  | average |  | Very Good |

**Table 3A: Global rating for scaling**
